# Monitoring quality in Israeli primary care: The primary care physicians' perspective

**DOI:** 10.1186/2045-4015-1-26

**Published:** 2012-06-20

**Authors:** Rachel Nissanholtz-Gannot, Bruce Rosen

**Affiliations:** 1 Smokler Center for Health Policy Research, Myers-JDC-Brookdale Institute, JDC Hill, POB 3886, Jerusalem, 91037, Israel; 2 Department of Health Management, Ariel University Center, Ariel, Israel; 3 See Author Information section at the end of the article for the full list of group members

## Abstract

**Background:**

Since 2000, Israel has had a national program for ongoing monitoring of the quality of the primary care services provided by the country's four competing non-profit health plans. Previous research has demonstrated that quality of care has improved substantially since the program's inception and that the program enjoys wide support among health plan managers. However, prior to this study there were anecdotal and journalistic reports of opposition to the program among primary care physicians engaged in direct service delivery; these raised serious questions about the extent of support among physicians nationally.

**Goals:**

To assess how Israeli primary care physicians experience and rate health plan efforts to track and improve the quality of care.

**Method:**

The study population consisted of primary care physicians employed by the health plans who have responsibility for the quality of care of a panel of adult patients. The study team randomly sampled 250 primary-care physicians from each of the four health plans. Of the 1,000 physicians sampled, 884 met the study criteria. Every physician could choose whether to participate in the survey by mail, e-mail, or telephone. The anonymous questionnaire was completed by 605 physicians – 69% of those eligible. The data were weighted to reflect differences in sampling and response rates across health plans.

**Main findings:**

The vast majority of respondents (87%) felt that the monitoring of quality was important and two-thirds (66%) felt that the feedback and subsequent remedial interventions improved medical care to a great extent. Almost three-quarters (71%) supported continuation of the program in an unqualified manner. The physicians with the most positive attitudes to the program were over age 44, independent contract physicians, and either board-certified in internal medicine or without any board-certification (i.e., residents or general practitioners). At the same time, support for the program was widespread even among physicians who are young, board-certified in family medicine, and salaried.

Many physicians also reported that various problems had emerged to a great or very great extent: a heavier workload (65%), over-competitiveness (60%), excessive managerial pressure (48%), and distraction from other clinical issues (35%). In addition, there was some criticism of the quality of the measures themselves. Respondents also identified approaches to addressing these problems.

**Conclusions:**

The findings provide perspective on the anecdotal reports of physician opposition to the monitoring program; they may well accurately reflect the views of the small number of physicians directly involved, but they do not reflect the views of primary care physicians as a whole, who are generally quite supportive of the program. At the same time, the study confirms the existence of several perceived problems. Some of these problems, such as excess managerial pressure, can probably best be addressed by the health plans themselves; while others, such as the need to refine the quality indicators, are probably best addressed at the national level. Cooperation between primary care physicians and health plan managers, which has been an essential component of the program's success thus far, can also play an important role in addressing the problems identified.

## Background

Health care systems around the world are increasingly making use of monitoring and reporting systems as a key tool for improving quality of care, and use of these systems is expected to grow further in the years ahead [1,2]. The support of front-line professionals is widely considered critical to the success of these efforts [3-6]. Nonetheless, the literature includes only a few studies of how health care providers perceive and experience quality monitoring [7-13]. This paper reports on the findings of a large-scale survey of physicians participating in Israel's system for monitoring the quality of community-based services. The Israeli system is of particular interest because it encompasses virtually all of the nation’s primary care physicians.

Israel's National Health Insurance (NHI) Law entitles all persons residing in the State of Israel to a broad package of health care benefits including hospital care, community-based services, and pharmaceuticals. Each resident is free to choose from among four non-profit, competing health plans. The plans are required to provide all members with the full range of benefits prescribed by the NHI law and, in return, receive a capitation payment from the government [14].

In 2000, Israel initiated a program to monitor the quality of the community-based services provided by the health plans. It is currently operated by the Ministry of Health as a national program under the auspices of the Israel National Institute for Health Policy. At present, approximately 40 quality indicators are used by the national program.^a^ They focus on the areas of immunizations, early detection of cancer, diabetes care,^b^ asthma, and cardiovascular care. All four health plans participate in the program and they receive financial support from the government to help defray the costs of monitoring and reporting on the quality indicators. However, the program does not offer financial incentives for high performance (P4P) to either health plans or individual physicians.

Two of the plans had actually begun independent quality monitoring programs prior to the initiation of the national program, and they subsequently integrated the two efforts. As a result, all the plans utilize the full set of indicators specified by the national program, and two of the plans also utilize a limited number of additional indicators.

The Israeli quality monitoring program drew heavily on the U.S. system for monitoring quality in health plans (HEDIS), in terms of both approach and specific measures. The unique features of the Israeli system include its coverage of the entire population, the extensive reliance on electronic health records, and a focus on a limited number of clinical areas [15]. A recent comparison of U.S. and Israeli performance on 11 roughly comparable measures found similar levels of quality overall, along with much more rapid rates of improvement in Israel, despite its much lower level of per capita health expenditure [16]. Several factors probably contribute to this more rapid rate of improvement, including the tighter working relationships between health plans and physicians in Israel [17]. Not surprisingly, the Israeli monitoring program enjoys wide support among health plan managers, who have implemented a broad range of interventions to improve performance as measured by the indicators [18].

While the support of front-line health care providers is a prerequisite for the success of quality improvement efforts, we know of only a few studies of practitioners' attitudes to, and perceptions of, quality monitoring systems. Almost all of them were qualitative and based on semi-structured interviews of a restricted number of practitioners. For example, a qualitative study of the attitudes of Canadian family physicians to accountability for quality revealed that they viewed the private feedback they received as a necessary part of medical professionalism; however, they were reluctant to share this feedback with patients [13]. A qualitative study of UK general practitioners revealed five types of concerns related to clinical performance indicators: the credibility of the indicators, their ulterior purpose, the growing need to demonstrate competence, perceptions of autonomy, and the identity of the assessor of their performance [10]. Another qualitative study of UK family physicians' attitudes to the NHS' quality monitoring/pay-for-performance scheme revealed that they believed it had changed physician behavior, that it had achieved targets in terms of improvements in disease-specific processes of care, and that aligning targets to professional priorities and values enhanced enthusiasm and understanding [8]. Other studies of UK general practitioners have similarly reported improvements in the organization, teamwork, consistency, and recording of care for conditions incentivized in the scheme, but not for non-incentivized conditions. Many respondents felt that the need to carry out and record specific clinical activities changed the emphasis away from patient-centered care. Doctors acknowledged improved disease management and teamwork but expressed unease about “box-ticking” and increased demands of team supervision, and some participants reported data manipulation to maximize practice income [12].

Other studies have focused on doctors' attitudes to practice support provided by health plans – an issue that is clearly related to quality monitoring, albeit not identical to it [7,9,11]. For example, a mail survey of 11,453 U.S. generalist and specialist physicians found that the practice-support strategies they found most useful were clinical guidelines and disease management programs, patient-specific drug information, reminders on patient health needs, reports on patient satisfaction, reports on the use of referrals or tests, and reports on immunization and mammography [9].

The objective of this study is to assess how Israeli primary care physicians experience and rate health-plan efforts to track and improve the quality of care. It was motivated, in part, by the need of Israeli policymakers to understand how the country succeeds in improving quality of community care and whether physician opposition constitutes a salient risk to the program. Prior to this study there were anecdotal and journalistic reports of opposition to the program among some primary care physicians [19]. These raised serious questions about the extent of support among physicians nationally, and the present study seeks to provide systematic and representative answers to those questions.

It was not possible for Israeli policymakers to rely solely on studies from abroad, in part because of the paucity of relevant quantitative studies. Moreover, there are significant differences in the organization of community care and in the quality monitoring programs between the U.S., Canada, and the UK on the one hand and Israel on the other, which preclude assuming that findings from one country are directly applicable to another. Therefore, we surveyed a national representative sample of Israeli primary care physicians who are front-line participants in the monitoring system with the objective of assessing how they experience and rate health-plan efforts to track and improve the quality of care.

## Methods

### Study population

The study population consisted of a stratified random sample of primary care physicians employed by the health plans. Physicians were included irrespective of whether they worked full time or part time, and whether they worked on a salaried basis or as independent contractors.^c^ As almost all quality indicators pertain to adult care, we excluded pediatricians. We also excluded physicians who do not have responsibility for the quality of care for a panel of patients: consultants, physicians who engaged mainly in administrative or managerial work, junior residents, retired physicians, and temporary replacements. The study team estimated that, at the time of the study, approximately 4,400 Israeli physicians met these criteria.^d^

### Study sample and data collection

Altogether, 1,000 physicians^e^ were randomly sampled – 250 from each of the four health plans. Of these, 884 met the study criteria. Pediatricians were generally excluded prior to sampling the 250 physicians from each plan. In contrast, physicians lacking responsibility for a panel of patients were only identified (and hence excluded from the study) at the time that they were contacted by project staff.

The survey was conducted between August and December 2010. Physicians were approached by mail with an explanation of the objectives of the survey and given the opportunity to refuse to participate. A week later, they were mailed the questionnaire and provided with a choice of responding by mail, e-mail, or telephone. About half responded by mail (53%), about a third used the telephone (33%), and the remaining 14% responded by e-mail.

### Response rate and weighting

605 of the physicians who met the study criteria – 69% of the sample – completed the questionnaire.^f^ The main reasons for non-response were refusal to participate in the survey (15%) and failure to locate (7%).^g^

The data were weighted to reflect the differences among the health plans in sampling ratios and response rates,^h^ so that the results would more accurately reflect the national study population. The weighting also took into account the relationship between the sampling probability and the number of health plans where each physician worked (i.e., a physician working for two health plans was more likely to be included in the sample than a physician working for only one plan).^i^

### The questionnaire

The main topics covered in the questionnaire were: (a) the doctors' experiences with the program; (b) their assessment of its impact on their work, patient care, and their relationship with their patients, their colleagues, and their health plans; (c) their perceptions of the quality indicators and their definitions; (d) how the physicians are using the information gathered through the program; (e) their difficulties and concerns regarding the quality assurance program; (f) their satisfaction with the program and their desires regarding its future; (g) their suggestions for improving the program; and (h) their personal and professional characteristics.

Decisions regarding the choice of topics, as well as the choice of specific questions and their formulation, were made through an iterative process involving the study team and the project steering committee, which consisted of many of Israel's leading experts on the quality monitoring system. While several questions were taken from other surveys (e.g., the Myers-JDC-Brookdale Institute's surveys of primary care physicians and specialist physicians) and adapted to meet the needs of this study, most of the questions were developed specifically for this study. The full questionnaire was piloted on a random sample of 10 primary care physicians and those questions found to be poorly understood were either modified or removed.

The final questionnaire included approximately 70 items. Many, though not all, of the questions that explored the extent of key phenomena used a six-point scale ranging from "not at all" to "very great". Generally speaking, in the tables and charts that follow we present the full distributions, while the text highlights the percentage of respondents who used the two high-end categories: "great" or "very great".

The questionnaire addressed the physicians' experience with the quality monitoring effort that they experienced in their health plans, and did not distinguish between the indicators common to all the plans as part of the national program and the additional indicators employed in two of the plans.

### Data analysis

This paper focuses on the findings for the physician population as a whole. In addition, various analyses were carried out to explore differences among various physician subgroups. These bivariate and multivariate analyses made use of the complex samples option of the Statistical Package for the Social Sciences (SPSS), Version 13. To analyze the responses to the open-ended questions, we used the SPSS facility that permits coding of multiple responses and quantitative assessment of the frequency of each response. Non-responses to the closed-ended questions were treated as missing values.

## Results

### Characteristics of the sample

As indicated in Table 1, the study sample was quite varied with regard to both personal characteristics (age, sex, population group, and country of birth) and professional characteristics (specialty and type of employment). As would be expected in an Israeli sample of physicians working in primary care, only a very small percentage (4%) works primarily as specialists.^j^

**Table 1:** Distribution of respondents by key personal and professional characteristics (percent)

**PERSONAL CHARACTERISTICS**		**PROFESSIONAL CHARACTERISTICS**	
**Age**		**Specialty**	
≤44	26	Family medicine	43
45–60	55	Internal medicine/other	19
>60	19	Not board certified	38
**Sex**		**Type of employment**^**#**^	
Female	44	Salaried only	48
Male	56	Independent only	25
		Both salaried and independent	26
**Population group**			
Non-Jew	24	**Main type of practice**	
Jew	76	As primary care MD	96
		As specialist MD	4
**Country of birth**			
Outside of Israel	60		
Israel	40		

# Total is less than 100 due to rounding.

The study team compared the characteristics of the respondents and the study population for Israel’s largest health plan, which accounts for about half of the physician population. The two were found to be very similar with regard to sex and whether they worked as independent contractors; the sample had a slightly higher concentration of young physicians up to age 44 (33% in the sample vs. 21% in the population) and board-certified specialists (73% vs. 60%). Similar comparisons were not carried out in the other three plans due to lack of available data on the physician population meeting study criteria.

### Perceptions of the monitoring program and of the quality indicators

The vast majority of respondents (87%) felt that the monitoring of quality was either important (50%) or very important (37%). Two-thirds of the respondents (66%) felt that the feedback and subsequent remedial interventions improved medical care to a great or very great extent. Almost three-quarters of respondents (71%) supported continuation of the program in an unqualified manner, and another 11% supported its continuation in part or under certain conditions.

There was widespread support of the monitoring program's choice of broad clinical areas, with 76% indicating that the areas were chosen appropriately to a great or very great extent. There was also a fair amount of support for the way the specific indictors had been defined, with 60% indicating that they were appropriately defined to a great or very great extent.

At the same time, about a third of respondents recommended that some of the specific indicators needed to be modified to a great or very great extent (Table 2). Ten percent of respondents identified specific clinical areas as unnecessary (e.g., vaccinations) and about half the respondents identified specific indicators as unnecessary. The indicators most often identified as unnecessary were the occult fecal blood test indicator and various indicators related to diabetes. Conversely, a number of respondents suggested adding indicators of the quality of communication with patients and of the care of mental disorders.

**Table 2:** Perceptions of the monitoring program and the quality of the indicators: Respondents’ assessments of the extent of related phenomena (percent)

	**Very great**	**Great**	**Moderate**	**Minor**	**Very minor**	**Not at all**
Feedback and remedial efforts improved quality^a^	20	46	24	7	3	1
The clinical areas selected were appropriate	14	62	21	2	1	0
The indicators were defined appropriately	8	51	35	4	2	0
The indicators need to be modified	9	23	27	19	8	14

^a^Total exceeds 100 due to rounding.

An overwhelming majority of the respondents (83%) agreed that patients' psycho-socioeconomic determinants affect clinical outcomes. A very large majority also felt that the use of the indicators should take into account the patients' psychosocial status (91%), socioeconomic status (73%), and general health (92%) to a great or very great extent (Chart 1).

**Figure 1:**
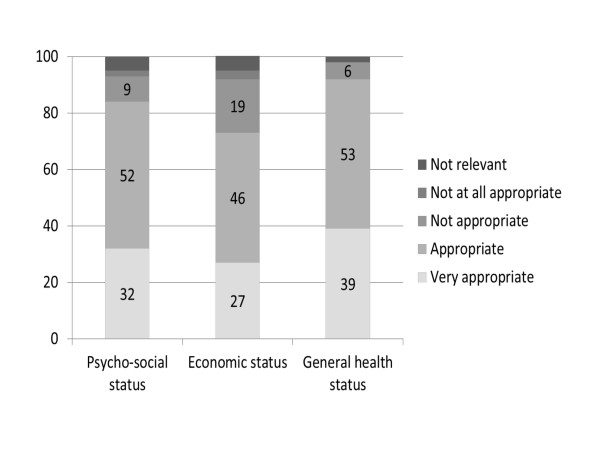
Extent to which respondents feel it would be appropriate to weight performance to reflect patient characteristics (percent).

When asked to suggest ways to improve the monitoring program and its implementation, about one-third of respondents provided specific suggestions. The suggestions proposed most often by the physicians were: reducing the number of indicators, adjusting the indicators to reflect the socioeconomic status of their patients, and hiring additional auxiliary staff; these were cited by 9%, 19%, and 9%, respectively, of the physicians who made specific suggestions.

### Doctors' relations with the health plans and other professionals

Most respondents (62%) had received explanations about the quality indicators from their health plans, and about two-thirds of that group thought that these explanations were adequate. Similarly, about half of the respondents (55%) indicated that they had received training from the plans on how to improve performance, and about two-thirds of that group found the training adequate. Half the respondents (50%) indicated that the health plans were doing everything they could to help physicians improve performance to a great or very great extent, and another 27% indicated that they did so to a moderate extent.

Almost all of the physicians (91%) reported that they received computerized reminders when they saw patients for whom the quality indicators suggested that additional health care was needed. Similarly, about three-quarters of the physicians (76%) reported that they periodically received a list of patients who had not been given the required checks or treatment.

There was a great deal of diversity in the extent to which respondents felt that the health plans were allowing them to deviate from the protocols embedded in the quality indicators in those cases where it made sense clinically to do so^k^; 42% indicated that they could do so to a great or very great extent, 33% indicated the ability to do so to a moderate extent, and 25% indicated that they could so only to a minor/very minor extent or not at all. At the same time, 37% of respondents indicated that the monitoring led to clinically unnecessary tests or treatments to a great or very great extent.

About half of the physicians (47%) felt that the program enhanced teamwork in the health plan to a great or very great extent (Table 3). As many as 80% agreed that nurses fully shared responsibility with them for what had been achieved through the program, and 60% felt that the nurses helped them to improve the quality of their practice procedures, as indicated in the monitoring program, to a great or very great extent.

**Table 3:** Physicians' relations with the health plans and other professionals: Respondents’ assessments of the extent of related phenomena (percent)

	**Very great**	**Great**	**Moderate**	**Minor**	**Very minor**	**Not at all**
The health plan is doing everything it can to help physicians improve their performance	11	39	27	16	4	3
The physician has the capacity to deviate from the protocol	10	32	33	18	4	3
The program has led to improved teamwork	8	39	24	13	7	9
Physicians assist one another with suggestions on how to improve performance	5	24	29	20	8	14
The feedback on performance has helped me become a better physician	9	40	29	11	5	6
The computerized reminders are helpful in patient care	24	46	19	8	2	1

There was a great deal of variety in the extent to which respondents endorsed the statement "Doctors help one another with suggestions on how to improve performance on the indicators"; 29% indicated that they do so to a great or very great extent, 29% indicated that they do so to a moderate extent, and 42% indicated that they do so to a minor/very minor extent or not at all (Table 3).^l^

### Effect on doctor-patient relations and on patient care

About half of the respondents (53%) felt their relationships with patients had improved as a result of the program, about two-fifths (38%) reported no change, and the remaining 9% felt that the relationship had deteriorated.

About two-thirds of the physicians who had received computerized reminders about patients who failed to adhere to quality standards stated that the reminders helped them provide better care to a great or very great extent.

Approximately two-thirds of our respondents (64%) reported that they generally begin an encounter by focusing on the patient's complaints (rather than soliciting information related to the quality indicators), and another 30% reported that they sometimes did so. Regarding the patient encounter as a whole, approximately one-third of the physicians (35%) claimed that adherence to quality standards distracted them from other clinical issues to a great or very great extent, and another 30% felt that this was the case to a moderate extent.

About half of the physicians (56%) reported that, in the wake of the quality monitoring effort, they had made changes in their practices. General changes included more testing and more thorough patient monitoring. Specific changes included those in referral, testing, counseling, and consultations of patients with diabetes; in outreach to women and explanations about the importance of mammograms; and in explanations and outreach to patients about vaccinations.

### Perceived problems associated with the program

Almost two thirds of the respondents (65%) indicated that their workload had increased to a great or very great extent as a result of the quality monitoring program. Almost as many (60%) indicated that there existed excess managerial pressure regarding the quality indicators. About half (48%) indicated feeling that there is over-competition regarding the indicators, to a great or very great extent (Chart 2).

**Figure 2:**
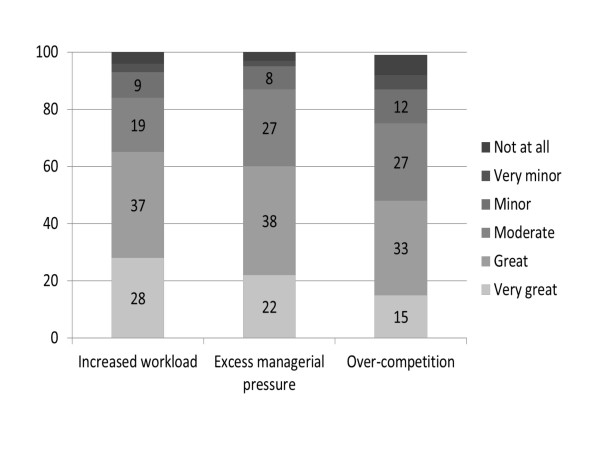
Extent to which respondents perceive the program to be associated with various problems (percent).

### Physician suggestions for addressing the problems and improving the program

Respondents who indicated that they felt over-competition to a great or very great extent were asked what changes, if any, should be made to reduce the over-competition; 27% of them provided specific suggestions, with the most common being not to make/publicize comparisons and to present the results in a different manner.

Respondents who indicated that they felt excess managerial pressure to a great or very great extent were similarly asked what changes, if any, should be made to reduce the pressure; 28% of them provided specific suggestions, with the most common being reducing the workload, providing more time for patient encounters, and changing managerial attitudes.

When asked to identify the main change they would make in how the health plans use the indicators, 29% of the full sample responded; the most common responses were allocating more time for each patient encounter, reducing the number of indicators, adjusting indicators to reflect patient socioeconomic status, reducing the workload (e.g., by adding support staff), and not using the indicators as a tool for pressuring the physicians or for promoting competition among them.

### Physicians' job satisfaction

Generally speaking, the physicians who participated in this survey were quite satisfied with their work; 80% indicated that they were generally satisfied or very satisfied with their practice, 15% were moderately satisfied, and 5% were unsatisfied or very unsatisfied. About half (46%) of the respondents reported an improvement in job satisfaction since the implementation of the monitoring program, about a third (32%) stated that the program had not affected their satisfaction, and about a fifth (22%) responded that the program had made them less satisfied with their work.

### Correlates and predictors of physicians' attitudes/perceptions

Bivariate analyses were used to examine how five of the study's main outcome variables varied across key subgroups of physicians. Three of these variables focused on perceived problems associated with the program (excessive managerial, pressure perceived overload, and over-competition), while the other two focused on positive dimensions (assessment of the program's contribution to quality, and support for its continuation). As indicated in Table 4, physicians who are either board-certified in internal medicine or without specialty board certification (i.e., general practitioners or residents), are over age 44 (and even more so for those over age 60), or are working as independent contractors, tended to have the most positive attitudes to the monitoring program. Yet, a high level of support for the program was also recorded among the physicians who were younger, salaried, and board-certified in family practice. On the other hand, even among physician sub-groups with the most positive attitudes to the program, a considerable percentage reported overload, over-competition, and excessive managerial pressure.

**Table 4:** The relationship between the main study variables and selected physician characteristics

	**Excessive managerial pressure**	**Excessive workload**	**Excessive competition**	**Contributes substantially to quality**	**Supports continuation**
**Total**	**60**	**65**	**48**	**66**	**71**
**Age**					
≤44	**65**	65	**51**	**57**	**68**
45–60	**60**	66	**50**	**70**	**72**
>60	**53**	64	**39**	**67**	**75**
**Sex**					
Female	**57**	**61**	**43**	66	71
Male	**62**	**69**	**51**	64	70
**Population group**					
Non-Jew	**65**	**69**	**61**	**77**	**82**
Jew	**58**	**64**	**44**	**62**	**67**
**Country of birth**					
Outside of Israel	**53**	**60**	**39**	66	**73**
Israel	**71**	**73**	**62**	65	**68**
**Specialty**					
Family medicine	**75**	**74**	**60**	**58**	**61**
Internal medicine/other	**45**	**58**	**34**	**68**	**73**
Not board certified	**50**	**58**	**40**	**73**	**81**
**Type of employment**					
Salaried only	**61**	**61**	**49**	**64**	**68**
Independent only	**47**	**58**	**38**	**71**	**76**
Both salaried and independent	**70**	**80**	**57**	**63**	**69**
**Main type of practice**					
As primary care MD	**61**	**66**	**49**	**65**	**70**
As specialist MD	**50**	**57**	**39**	**76**	**91**
**Response mode**					
Regular mail	**62**	66	**45**	**59**	**64**
E-mail	**50**	62	**38**	**55**	**60**
Telephone	**61**	65	**59**	**82**	**87**

(extent to which each outcome was felt to a great or very great extent – in percent).

Note: Contrasts found to be significant at the .05 level appear in bold.

Table 5 presents the results of logistic regressions that were undertaken to assess how those same five outcome variables were independently affected by physicians' personal and demographic characteristics (age, country of birth, ethnic group, gender, clinical specialty, employment status) as well as the survey response method. Being over age 45, female, non-Israeli born, non-Jewish, and without board-certification (i.e., being a general practitioner or a resident) were all found to have independent positive effects on attitudes to the monitoring program, as were working primarily as a specialist and working as an independent only. Confidence intervals for all the independent variables, for all five equations, can be found in the Additional file 1: Appendix tables.

**Table 5:** Logistic regressions of selected outcome variables on physicians' personal and professional characteristics*

	**Excessive managerial pressure**	**Excessive workload**	**Excessive competition**	**Contributes substantially to quality**	**Supports continuation**
**Age** (Reference group: Age < 45)					
45–60	1.0	**1.6**	1.1	**1.3**	0.9
≥61	**1.4**	**1.5**	**0.6**	**0.8**	**0.5**
**Born in Israel**	**0.8**	0.9	**2.1**	1.0	**1.8**
**Jewish**	**0.5**	**0.5**	1.1	1.0	**1.4**
**Male**	**0.7**	**0.6**	1.1	1.2	**1.3**
**Board certification** (Reference group: Not board certified)					
Family physician	**0.6**	**0.6**	1.0	0.9	**1.2**
Internist and other	**0.5**	**0.7**	**0.6**	0.8	**0.6**
**Work primarily as specialist**	**6.1**	**2.2**	0.9	**0.7**	0.8
**Mode of employment** (Reference group: Independent only)					
Salaried only	**1.3**	0.9	**1.3**	**0.7**	1.2
Salaried and independent	**1.5**	**0.7**	1.2	**1.5**	**1.4**
**Response mode** (Reference group: Telephone)					
Regular mail	**0.4**	**0.4**	**0.5**	**1.2**	1.1
E-mail	**0.3**	**0.3**	**0.5**	0.9	**0.6**
					
					
					
Cox & Snell R^2^	0.17	0.12	0.18	0.18	0.21
Nagelkerke R^2^	0.24	0.16	0.25	0.25	0.28
N (unweighted)	556	552	557	554	557

*The set of independent variables also included the main health plan with which the physician worked. However, the coefficients of the health plan variables are not presented here, in keeping with the health plans' conditions for participating in the study.

Coefficients in bold were significant at the .05 level.

Table 6 presents the results of a logistic regression undertaken to assess how support for continuation of the quality monitoring programs was affected by a variety of perceived program effects, which were treated as intermediate outcomes. As expected, a perception that the quality standards were valid and perceptions that the program improved patient care, teamwork, doctor patient relations, and doctors' job satisfaction, were all independently associated with support for the continuation of the monitoring program.

**Table 6:** Results of logistic regression to explore the independent effects of perceived program impacts on support for program continuation*

	
Excessive workload	**0.4**
Excessive competition	1.0
Excessive managerial pressure	**0.7**
All, or almost all, indicators defined appropriately	**2.6**
Improved quality	**3.8**
Enhanced teamwork	**2.1**
Improved relationships with patients	**2.6**
Improved work satisfaction	**2.5**
	
	
	
Cox & Snell R^2^	0.36
Nagelkerke R^2^	0.52
N (unweighted)	541

## Discussion

We performed the first survey of physicians’ perspectives on Israel’s quality monitoring system. Two main findings emerge from the present survey. First, the vast majority of the physicians perceived the quality monitoring program as contributing positively and substantially to patient care, and most physicians supported the continuation of the program. They also indicated, by a 2 to 1 margin, that the program had increased their work satisfaction. Physicians' widespread support is remarkable in light of the enormity of the changes that accompanied the introduction of Israel's national quality assurance program. The survey finding of widespread support contrasts with the impression that had been created by a limited number of highly visible objections.

Second, physicians reported six main problems that they associated with the program: increased workload, over-competition, excessive managerial pressure, distraction from other clinical issues, concerns about the validity of some of the quality standards, and encouragement of unnecessary tests or treatments. To be sure, it is hard to imagine that a quality monitoring system could be implemented without some increase in workload and managerial pressure. Still the large percentages of physicians reporting over-competition and excessive managerial pressure are definitely worthy of attention and may also be ameliorable.

### Comparisons with other countries

These findings are consistent with those of qualitative studies in the UK and North America. Specifically, they support the association between doctors' perceptions of quality monitoring and the validity of the quality standards [7,12]. Our findings also support those of other surveys indicating that doctors believe that quality monitoring has improved disease-specific processes of care [8], as well as health care organization and teamwork [12].

On the other hand, when compared to their counterparts abroad, Israeli physicians appear to be more likely to consider computerized reminders effective, and less likely to perceive performance indicators as detrimental to physician-patient relations. For example, the qualitative studies carried out among UK physicians found that various quality monitoring efforts (some of which were accompanied by pay for performance incentives) may have eroded doctor–patient relations and reduced doctors' focus on patients' complaints. In contrast, only 10% of Israeli physicians reported that quality monitoring (in the absence of P4P incentives) had eroded their relationships with patients and 50% even reported improved relationships. Furthermore, the preponderance of our respondents reported that they usually begin a patient encounter by focusing on the patient's presenting complaints.

### Reflections on selected findings

The study identified the following characteristics as being independently associated with above average levels of support for the monitoring program: being over age 45, female, non-Israeli born, non-Jewish, and/or without board certification as well as working as an independent contractor and/or primarily as a specialist. The present study does not provide any empirical data on the reasons behind these associations. Possible explanations could relate to such factors as the extent to which each group is exposed to managerial oversight on a day-to-day basis, has confidence in its professional capabilities and status in the system, has achieved improvements on the quality indicators, engages in ongoing CME activities, and has been culturally or socially influenced to be open to standards, monitoring, and supervision. It would be informative to test the existence and power of these factors in future research.

Some readers of the article may be surprised or even puzzled by the findings that many physicians report that the quality monitoring/improvement effort has improved their work satisfaction and their relationships with their patients. With regard to work satisfaction, it may be that, for many physicians, the disadvantages in terms of increased workload and managerial pressures are outweighed by the advantages of having clearer guidance from their employers about what is expected of them, receiving from their employers additional resources and tools for improving quality, seeing improvements in their performance on the quality measures along with recognition for those improvements, etc. This, too, could be the subject of future research and, in this case, our own database can probably be mined further to generate at least partial answers to these questions.

With regard to relationships with patients, it may be that many physicians have figured out how to successfully integrate the need to respond to patients' presenting complaints with the pro-active encouragement of various screening tests and behavioral changes emphasized by the indicators. Moreover, it may be that many patients appreciate a proactive approach on the part of their personal physicians and interpret it as concern and professionalism. Of course, there are probably additional possible explanations, as well as a great deal of variation on this issue among both patients and physicians. Clearly, this is an also a fertile area for further research.

The finding that about one-third of the physicians perceive the monitoring program as promoting unnecessary tests or treatments should concern health plan executives. If possible, this perception should be tested empirically, and efforts should be made to quantify the cost impact of any program-generated unnecessary tests and identify their causes. It may be that imprecision in the definition of the target populations for some of the indicators are contributing to unnecessary testing. If so, any data on the cost impact would give further impetus to efforts to refine these definitions.

It is somewhat disheartening that only about one-third of the respondents report that physicians provide suggestions to one another on how to improve performance on the indicators to a great or very great extent. This may be related, in part, to the high prevalence of reports of "over-competition". Other factors may include insufficient time to provide suggestions due to the work burden, the paucity of opportunities to observe the work of peers in clinic settings, and a culture of non-interference. It might be useful for the health plans to explore further the reasons for the limited peer input and to seek ways to expand it.

### Study limitations

The study is limited by the lack of full comparative data regarding the characteristics of the sample and the population. In the largest health plan, we found that the sample contained a higher concentration of board-certified and younger physicians than did the population – two groups that tend to be less supportive of the program than the average. If a similar situation obtains in the other health plans, the results presented in this paper may constitute an underestimate of the extent of primary care physician support of the monitoring program.

It is also possible that the physicians' perceptions of certain program effects (e.g., their reports of increased workload as a result of the quality monitoring) may have been influenced somewhat by exogenous and unrelated health system changes. For example, between 2000 and 2010 the number of Israeli physicians up to age 65 per 1,000 population dropped from 3.7 to 3.4 and this may well have affected the workloads of community-based physicians. In addition, over the past decade the health plans have also increased the responsibilities of the physicians in many areas unrelated to the quality monitoring program.^m^

On the other hand, it is important to note that the study took place during a period when responsibility for the monitoring project at the national level was being shifted from one university to another. During this period, efforts to improve performance within the health plans reportedly continued unabated. Still, the absence of changes in the number of indicators and their definitions emanating from the national level may have temporarily reduced the amount of work generated by the program for primary care physicians.

A final study limitation is that the analyses do not distinguish among health plans, as the health plans' agreement to participate in the study was conditioned on a commitment from the study team not to publish plan-specific findings. Thus, the study does not include an analysis of the relationships between plan-specific factors (such as the number of indicators monitored and how they were used managerially) and key outcome variables (such as increased workload and excess managerial pressure).

### Strategies for strengthening the quality monitoring program

Addressing the perceived problems of the Israeli quality-monitoring program may further increase the physicians' motivation in promoting quality, strengthen their support of the program, and contribute to its continuation and further development. Some of these problems, such as excess managerial pressure, can probably best be addressed by the health plans themselves, while others, such as the need to refine the quality indicators, are probably best addressed at the national level.

As indicated in the findings section, the physicians were given several opportunities in the questionnaire to suggest ways to improve the quality monitoring program and address the problems that had arisen. The recommendations cited relatively frequently included:

· Re-defining or deleting some of the quality standards, such as body mass index.^n^

· Considering differences in the patient mix when evaluating performance and providing feedback to individual physicians.

· Reducing perceived over-competition and managerial pressure, for example, by reconsidering the frequency and prominence of the performance comparisons among physicians.

· Exploring ways to reduce workload, such as expanding the staff that assists the physicians.

· Increasing the time allotted for patients' visits, especially in the case of patients with chronic diseases.

While implementing either of the latter two proposals on a nationwide basis would no doubt be quite costly, health plan executives are encouraged to give them due consideration, particularly in light of the findings of this survey.

In addition, it would appear to us that, in light of the study findings, health plans and the national project team might consider the following:

· Strengthening awareness of the positive aspects of the program, such as the contribution of the program to teamwork and quality of care.

· Proceeding cautiously when adding new indicators, and assessing the trade-off between the benefits of adding indicators (e.g., the potential to improve quality in additional clinical domains) and its costs (e.g., increased doctors' workload).

Improvements in quality of care are often the product of the combined efforts of health plan managers and health care professionals engaged in direct service delivery. Accordingly, the study reported here, which examined how primary care physicians perceive the program and how it has affected their practices, was part of a larger, two-part study. The first part of the study used in-depth interviews to examine how managers in the health plans perceived the quality-monitoring program and what they did in order to translate its results into improved quality [19]. One of the main findings of that part of the study was that physicians and health plan managers were working closely together to turn data on quality into quality improvements, a finding corroborated by various in-depth studies focusing on particular health plans [21,22]. A related study suggested that these close working relationships might be one of the reasons why performance on the quality indicators is improving more rapidly in Israel than in the U.S. [17].

It will be important for physicians and health plan managers to continue to work together, along with the leadership of the national quality monitoring program, in planning for the forthcoming release of plan-specific performance data. Currently, health care performance data are publicly available at the national level only, and the present study did not address the question of whether it is desirable to publish performance by health plan. In May 2011, the Jerusalem District Court ruled in favor of publishing performance data by individual health plans. Our findings suggest that implementation of this decision be handled cautiously by all involved – the national program, the plans, and the physicians. This is because, while publication of plan-specific data may provide consumers with important information, it may also reduce the spirit of cooperation among the parties, which has contributed so much to the program's success to date [15,17]. It could also lead to further increases in the already high levels of managerial pressure on physicians to improve performance.

In this context it is worth noting that, in a recent IJHPR article, Jaffe and colleagues reported continued overall improvements in the quality indicators in the 2007–2009 period [23]. In a commentary on that article, Mark Chassin raised the question of "How good is good enough?" and encouraged Israeli health care leaders to strive for yet further gains in the quality of community-based health care [24]. Our study suggests that the extent to which Israeli primary care physicians would support enhanced efforts to improve quality could depend on the extent to which the program directorate and the health plans address concerns about indicator validity, excess managerial pressure, and the workload.

## Conclusion

### Possible lessons for other countries and topics for further research

As one of the world's first large scale surveys of health care professionals' perceptions of quality monitoring, this study makes a contribution that goes beyond the specific Israeli context by identifying issues that should be explored in other countries as well. Moreover, the correlates of Israeli physicians' support for the quality monitoring may be relevant for other countries. For example, our finding that excess managerial pressure and lack of confidence in the quality measures may erode physician support for monitoring systems may be generalizable. Similarly, some of the features of the Israeli health care system that appear to contribute to physician support of the quality monitoring program may be relevant to other countries as well. As reported recently [15], when compared with the U.S., the Israeli quality monitoring program focuses on a smaller number of clinical domains, a greater reliance on electronic health records, and a closer working relationship between physicians and health plans. The relationship between some of these features and physicians' perceptions of quality monitoring may be worth exploring.

In future studies, it would be worthwhile to investigate why physicians in Israel, unlike their colleagues in the U.S., find computerized follow-up more useful and why fewer Israeli physicians report that performance measures are detrimental to their relationships with patients. Future studies may also attempt to find out whether Israeli physicians are concerned that clinical guidelines and performance management limit their clinical autonomy and judgment similarly to UK [10] and U.S. [11] physicians. Future research might also assess the extent to which some of the intriguing findings of studies from abroad (such as the reported contribution of performance measures to physician accountability to patients and funders [13]) also obtain in Israel.

Another issue for further exploration in Israel is why some subgroups of physicians (such as young physicians) are less supportive than others of the quality monitoring effort and what could be done to increase their level of support. Similarly, it would be interesting to explore how various dimensions of the physicians' workload (such as the number of patients seen per hour) influence their level of support for the monitoring program. Finally, it will be important to repeat the current study in a few years, to assess whether and how the patterns of physician support for quality monitoring change in the wake of public reporting of plan-specific performance data.

## End notes

^a^ Some of the health plans also use additional indicators that they have developed in-house.

^b^ Here, and elsewhere in the article, we use "diabetes" to refer to diabetes mellitus.

^c^ As indicated in Table 1, 48% of the primary care physicians surveyed as part of this study work are salaried physicians only, 25% are independent contractors only, and 26% work in both modalities. Study data also indicate that among those physicians working only as independent contractors, 76% work with only one health plan, 17% work with two plans, and 7% work with three or four plans [20].

^d^ The numbers of physicians in each health plan estimated to meet the criteria were as follows: 2,166 in Clalit, 1,059 in Maccabi, 835 in Meuhedet, and 857 in Leumit. These figures total 4,971, which is greater than 4,400 cited in the text for the country as a whole. The source of the discrepancy is that approximately 7% of the physicians work in 2 plans and approximately 2% work in 3 plans.

^e^ This sample size was chosen to enable identification of differences of 11 percentage points between key subgroups of physicians on the main outcome variables (e.g., the extent of support for continuation of the program), with an alpha of .05 and a beta of .80.

^f^ Factors contributing to the high response rate include: the existence of a stable, professionally trained, fieldwork unit with the Myers-JDC-Brookdale Institute, intensive efforts on the part of the fieldwork staff to obtain correct physician phone numbers in cases where the numbers originally supplied by the health plans proved to be incorrect, the policy of persistent and repeated attempts to reach all potential respondents over a period of several months and at various times of day (as needed), and the referral of potential refusals to the fieldwork supervisor or the investigators for special treatment.

^g^ It may be that some of the non-respondents have characteristics that exclude them from the study population; if so, the true response rate would be higher than the 69% reported here.

^h^ Health plan-specific response rates ranged from 53% to 77%.

^i^ Note that the weighting procedure did change the results, but only to a minimal extent.

^j^ Apparently, 4% of respondents work primarily as specialists, but also work secondarily as primary care physicians.

^k^ Even in the absence of financial incentives, health plans have a number of ways to communicate their wishes to physicians working with them on either a contractual or salaried basis, and of encouraging the physicians to practice in accord with those wishes. It appears that there are differences both between and within plans in the extent to which managers insist that physicians follow protocols in situations where the physicians have questions about whether the protocols are clinically appropriate.

^l^ Interestingly, the percent of respondents indicating that physicians provide suggestions to one another on how to improve performance to a great or very great extent did not differ much between those working primarily as salaried workers vs. those working primarily as independents (27% v. 25%). Similarly, that percentage was only slightly higher among physicians who work in settings with other physicians vs. those working alone (33%. vs. 27%). More substantial differences were related to whether the respondent worked primarily in a health plan-owned setting (29% vs. 16%).

^m^ The new responsibilities include improving patients’ electronic medical records, risk management, improving patient satisfaction with services, and inclusion of physicians in the activities related to member recruitment and retention.

^n^ Indeed, various efforts are already underway, at both the health plan and national levels, to refine some of the indicators. To date, the foci of these efforts have included refining the definition of the denominator population for the indicators used to monitor whether persons with asthma and certain types of heart disease are being treated appropriately.

## Competing interests

The authors declare that they have no competing interests.

## Authors' contributions

RN and BR were jointly responsible for the study design, data collection, data analysis, and writing of the article; each contributed intensively to all phases of the study. All members of the Quality Monitoring Study Group provided important input into the study design and data analysis, and commented on drafts of the article and/or the study report on which it is based. Eliana Meirowitz Nelson contributed to the literature review. All authors have read and approved the final version of the manuscript.

## Author information

Rachel Nissanholtz-Gannot is a researcher at the Smokler Center for Health Policy Research and a lecturer at the Ariel University Center.

Bruce Rosen is the Director of the Smokler Center for Health Policy Research at the Myers-JDC-Brookdale Institute, as well as co-editor of the IJHPR.

The members of the Quality Monitoring Study Group, and their positions at the time of the study, were as follows:

· Alik Aviram (Scientific Director, Israel National Institute for Health Policy Research)

· Arnon Cohen (Director of the Department of Quality Measures and Research, Chief Physician Office at Clalit Health Services)

· Tuvia Horev (Deputy Director-General for Health Economics and Insurance, Ministry of Health)

· Boaz Lev (Vice Director-General, Ministry of Health)

· Gordon Littman (Chairman of the Committee for Quality Improvements of the Association of Family Physicians in Israel)

· Ziva Litvak (Administrative Director, Israel National Institute for Health Policy Research)

· Eran Matz (Director of the Division of Clinical Medicine at the Leumit Health Fund)

· Eliana Meirowitz Nelson (Health Policy Fellow, Myers-JDC-Brookdale Institute)

· Joseph Rosenblum (CIO of the Medical Division of the Meuhedet Health Fund)

· Amir Shmueli (Professor of health economics at the School of Public Health, Hebrew University – Hadassah Faculty of Medicine)

· Hava Tabenkin, (Director of Family Medicine in the Northern Region, Clalit Health Services)

· Rachel Wilf-Miron (Director of Quality Management at Maccabi Healthcare Services)

· Shlomo Vinker (Chairman of the Association of Family Physicians in Israel)

ADC, JKR, EM, and RW-M are also members of the steering committee of the National Program for Quality Indicators in Community Healthcare in Israel. AS is also a member of the program directorate.

## Supplementary Material

Additional file 1 **Appendix Table 5A.** Logistic regression of "Supports Continuation" on physicians' personal and professional characteristics, including 95% confidence intervals*. **Table 5B:** Logistic regression of "Contributes Substantially to Quality" on physicians' personal and professional characteristics, including 95% confidence intervals*. **Table 5C:** Logistic regression of "Excessive Competition" on physicians' personal and professional characteristics, including 95% confidence intervals*. **Table 5D:** Logistic regression of "Excessive Workload" on physicians' personal and professional characteristics, including 95% confidence intervals*. **Table 5E:** Logistic regression of "Excessive Managerial Pressure" on physicians' personal and professional characteristics, including 95% confidence intervals*. Click here for file
