# Supplementary material for: Monitoring quality in Israeli primary care: The primary care physicians' perspective
Source: Isr J Health Policy Res. 2012 Jun 20;1:26. doi: 10.1186/2045-4015-1-26 (PMC3472172; doi:10.1186/2045-4015-1-26)
Supplement: Additional file 1 — Appendix Table 5A. Logistic regression of "Supports Continuation" on physicians' personal and professional characteristics, including 95% confidence intervals*. Table 5B: Logistic regression of "Contributes Substantially to Quality" on physicians' personal and professional characteristics, including 95% confidence intervals*. Table 5C: Logistic regression of "Excessive Competition" on physicians' personal and professional characteristics, including 95% confidence intervals*. Table 5D: Logistic regression of "Excessive Workload" on physicians' personal and professional characteristics, including 95% confidence intervals*. Table 5E: Logistic regression of "Excessive Managerial Pressure" on physicians' personal and professional characteristics, including 95% confidence intervals*. [file 2045-4015-1-26-S1.doc]

**Appendix Table 5A: Logistic regressions of "Supports Continuation" on physicians' personal and professional characteristics, including 95% confidence intervals***

Note: Coefficients highlighted in bold were significant at the .05 level

| 95% confidence intervals | |  |  |
| --- | --- | --- | --- |
| Lower bound | Upper bound | Point estimate |  |
|  | | | |
| **Age** (Reference group: Age under 45) | | | |
| 0.8 | 1.2 | 1.0 | Age 45-60 |
| 1.1 | 1.8 | **1.4** | Age 61+ |
|  |  |  |  |
| 0.6 | 0.9 | **0.8** | **Born in Israel** |
|  |  |  |  |
| 0.4 | 0.6 | **0.5** | **Jewish** |
|  |  |  |  |
| 0.6 | 0.9 | **0.7** | **Male** |
|  |  |  |  |
| **Board certification** (Reference group: Not board certified) | | | |
| 0.5 | 0.8 | **0.6** | Family physician |
| 0.4 | 0.7 | **0.5** | Internist and other |
|  |  |  |  |
| 3.5 | 10.8 | **6.1** | **Works primarily as specialist** |
|  |  |  |  |
| **Mode of employment** (Reference group: Independent only) | | | |
| 1.0 | 1.6 | **1.3** | Salaried only |
| 1.2 | 2.0 | **1.5** | Salaried and independent |
|  |  |  |  |
| **Response mode** (Reference group: Telephone) | | | |
| 0.3 | 0.5 | **0.4** | Regular mail |
| 0.2 | 0.3 | **0.3** | E-mail |
|  |  |  |  |
|  |  | 0.17 | Cox & Snell R^2^ |
|  |  | 0.24 | Nagelkerke R^2^ |
|  |  | 556 | N (unweighted) |

The set of independent variables also included the main health plan with which the physician worked. However, the coefficients of the health plan variables are not presented here, in keeping with the health plans' conditions for participating in the study.

**Appendix Table 5B: Logistic regressions of "Contributes Substantially to Quality" on physicians' personal and professional characteristics, including 95% confidence intervals***

Note: Coefficients highlighted in bold were significant at the .05 level

| 95% confidence intervals | |  |  |
| --- | --- | --- | --- |
| Lower bound | Upper bound | Point estimate |  |
|  | | | |
| **Age** (Reference group: Age under 45) | | | |
| 1.4 | 2.0 | **1.6** | Age 45-60 |
| 1.2 | 2.0 | **1.5** | Age 61+ |
|  |  |  |  |
| 0.7 | 1.1 | 0.9 | **Born in Israel** |
|  |  |  |  |
| 0.4 | 0.6 | **0.5** | **Jewish** |
|  |  |  |  |
| 0.5 | 0.7 | **0.6** | **Male** |
|  |  |  |  |
| **Board certification** (Reference group: Not board certified) | | | |
| 0.5 | 0.8 | **0.6** | Family physician |
| 0.6 | 0.9 | **0.7** | Internist and other |
|  |  |  |  |
| 1.5 | 3.3 | **2.2** | **Works primarily as specialist** |
|  |  |  |  |
| **Mode of employment** (Reference group: Independent only) | | | |
| 0.7 | 1.1 | 0.9 | Salaried only |
| 0.6 | 0.9 | **0.7** | Salaried and independent |
|  |  |  |  |
| **Response mode** (Reference group: Telephone) | | | |
| 0.3 | 0.4 | **0.4** | Regular mail |
| 0.2 | 0.4 | **0.3** | E-mail |
|  |  |  |  |
|  |  | 0.12 | Cox & Snell R^2^ |
|  |  | 0.16 | Nagelkerke R^2^ |
|  |  | 552 | N (unweighted) |

The set of independent variables also included the main health plan with which the physician worked. However, the coefficients of the health plan variables are not presented here, in keeping with the health plans' conditions for participating in the study.

**Appendix Table 5C: Logistic regressions of "Excessive Competition" on physicians' personal and professional characteristics, including 95% confidence intervals***

Note: Coefficients highlighted in bold were significant at the .05 level

| 95% confidence intervals | |  |  |
| --- | --- | --- | --- |
| Lower bound | Upper bound | Point estimate |  |
|  | | | |
| **Age** (Reference group: Age under 45) | | | |
| 0.9 | 1.3 | 1.1 | Age 45-60 |
| 0.5 | 0.8 | **0.6** | Age 61+ |
|  |  |  |  |
| 1.7 | 2.5 | **2.1** | **Born in Israel** |
|  |  |  |  |
| 0.9 | 1.4 | 1.1 | **Jewish** |
|  |  |  |  |
| 0.9 | 1.3 | 1.1 | **Male** |
|  |  |  |  |
| **Board certification** (Reference group: Not board certified) | | | |
| 0.8 | 1.2 | 1.0 | Family physician |
| 0.5 | 0.7 | **0.6** | Internist and other |
|  |  |  |  |
| 0.6 | 1.3 | 0.9 | **Works primarily as specialist** |
|  |  |  |  |
| **Mode of employment** (Reference group: Independent only) | | | |
| 1.1 | 1.7 | **1.3** | Salaried only |
| 0.9 | 1.5 | 1.2 | Salaried and independent |
|  |  |  |  |
| **Response mode** (Reference group: Telephone) | | | |
| 0.5 | 0.7 | **0.5** | Regular mail |
| 0.3 | 0.6 | **0.5** | E-mail |
|  |  |  |  |
|  |  | 0.18 | Cox & Snell R^2^ |
|  |  | 0.25 | Nagelkerke R^2^ |
|  |  | 557 | N (unweighted) |

The set of independent variables also included the main health plan with which the physician worked. However, the coefficients of the health plan variables are not presented here, in keeping with the health plans' conditions for participating in the study.

**Appendix Table 5D: Logistic regressions of "Excessive Workload" on physicians' personal and professional characteristics, including 95% confidence intervals***

Note: Coefficients highlighted in bold were significant at the .05 level

| 95% confidence intervals | |  |  |
| --- | --- | --- | --- |
| Lower bound | Upper bound | Point estimate |  |
|  | | | |
| **Age** (Reference group: Age under 45) | | | |
| 1.1 | 1.5 | **1.3** | Age 45-60 |
| 0.6 | 1.0 | **0.8** | Age 61+ |
|  |  |  |  |
| 0.8 | 1.2 | 1.0 | **Born in Israel** |
|  |  |  |  |
| 0.8 | 1.3 | 1.0 | **Jewish** |
|  |  |  |  |
| 1.0 | 1.4 | 1.2 | **Male** |
|  |  |  |  |
| **Board certification** (Reference group: Not board certified) | | | |
| 0.7 | 1.1 | 0.9 | Family physician |
| 0.7 | 1.1 | 0.8 | Internist and other |
|  |  |  |  |
| 0.5 | 1.0 | **0.7** | **Works primarily as specialist** |
|  |  |  |  |
| **Mode of employment** (Reference group: Independent only) | | | |
| 0.6 | 0.9 | **0.7** | Salaried only |
| 1.2 | 2.0 | **1.5** | Salaried and independent |
|  |  |  |  |
| **Response mode** (Reference group: Telephone) | | | |
| 1.0 | 1.4 | **1.2** | Regular mail |
| 0.7 | 1.2 | 0.9 | E-mail |
|  |  |  |  |
|  |  | 0.18 | Cox & Snell R^2^ |
|  |  | 0.25 | Nagelkerke R^2^ |
|  |  | 554 | N (unweighted) |

The set of independent variables also included the main health plan with which the physician worked. However, the coefficients of the health plan variables are not presented here, in keeping with the health plans' conditions for participating in the study.

**Appendix Table 5E: Logistic regressions of "Excessive Managerial Pressure" on physicians' personal and professional characteristics, including 95% confidence intervals***

Note: Coefficients highlighted in bold were significant at the .05 level

| 95% confidence intervals | |  |  |
| --- | --- | --- | --- |
| Lower bound | Upper bound | Point estimate |  |
|  | | | |
| **Age** (Reference group: Age under 45) | | | |
| 0.8 | 1.1 | 0.9 | Age 45-60 |
| 0.4 | 0.7 | **0.5** | Age 61+ |
|  |  |  |  |
| 1.5 | 2.2 | **1.8** | **Born in Israel** |
|  |  |  |  |
| 1.1 | 1.8 | **1.4** | **Jewish** |
|  |  |  |  |
| 1.1 | 1.5 | **1.3** | **Male** |
|  |  |  |  |
| **Board certification** (Reference group: Not board certified) | | | |
| 1.0 | 1.5 | **1.2** | Family physician |
| 0.5 | 0.8 | **0.6** | Internist and other |
|  |  |  |  |
| 0.5 | 1.1 | 0.8 | **Works primarily as specialist** |
|  |  |  |  |
| **Mode of employment** (Reference group: Independent only) | | | |
| 1.0 | 1.4 | 1.2 | Salaried only |
| 1.1 | 1.7 | **1.4** | Salaried and independent |
|  |  |  |  |
| **Response mode** (Reference group: Telephone) | | | |
| 0.9 | 1.3 | 1.1 | Regular mail |
| 0.5 | 0.7 | **0.6** | E-mail |
|  |  |  |  |
|  |  | 0.21 | Cox & Snell R^2^ |
|  |  | 0.28 | Nagelkerke R^2^ |
|  |  | 557 | N (unweighted) |

The set of independent variables also included the main health plan with which the physician worked. However, the coefficients of the health plan variables are not presented here, in keeping with the health plans' conditions for participating in the study.
